# A Survey of the Current Practice for Testing for Neonatal Hypoglycaemia in Aotearoa, New Zealand

**DOI:** 10.1111/jpc.70097

**Published:** 2025-05-31

**Authors:** C. M. Ulyatt, J. E. Harding, V. Clapham, J. M. Alsweiler, L. Lin

**Affiliations:** ^1^ Liggins Institute University of Auckland Auckland New Zealand; ^2^ New Zealand College of Midwives Te Kāreti o Ngā Kaiwhakawhānau Ki Aotearoa Christchurch New Zealand; ^3^ Department of Paediatrics: Child and Youth Health University of Auckland Auckland New Zealand; ^4^ Neonatal Intensive Care Unit Auckland City Hospital Auckland New Zealand

**Keywords:** diagnostic techniques and procedures, hypoglycaemia, infant, neonatal screening, newborn, surveys and questionnaires

## Abstract

**Aims:**

Neonatal hypoglycaemia is the most common metabolic disturbance in newborns and can lead to brain injury. However, the feasibility of implementing a national recommendation to use accurate testing methods to avoid under‐ and over‐diagnosis depends on current practise, preferences, and resources. We therefore sought to identify current testing practises for neonatal hypoglycaemia to inform recommendations for testing, including devices and timing.

**Methods:**

An online survey was sent to relevant clinical unit leaders across the 19 health regions of New Zealand, and to members of the New Zealand College of Midwives who provide home birth services. These individuals were asked to complete the survey themselves or identify the appropriate practitioner in their unit to complete it.

**Results:**

From 28th August to 12th December 2023, we obtained 71 responses, which came from more than one profession from all but one health region. The most commonly used devices for measuring glucose concentrations in capillary blood samples were blood gas analysers (20/65, 31%) and i‐STAT (12/65, 18%). Devices used did not differ among professions, but the use of blood gas analysers was not reported in primary units. i‐STAT (22/80, 28%) and blood gas analyser (19/80, 24%) were also the most preferred testing devices. The timing of blood sampling was reported to be similar for > 70% of respondents.

**Conclusions:**

Most healthcare professionals use and prefer accurate blood sampling devices, suggesting implementing a recommendation to use these is likely feasible. However, guidance on the timing of testing is needed.

**Summary**

- What is already known about this topic
  ○ Neonatal hypoglycaemia is common and can cause brain injury, so accurate testing is essential.
  ○ There is variation in clinical practise for managing neonatal hypoglycaemia across different hospitals and countries, suggesting that developing and implementing an evidence‐based national guideline may be helpful.
  ○ Information about current practises in testing for neonatal hypoglycaemia would help inform guideline recommendations that are likely to be feasible and implemented.
- What this study adds
  ○ Most practitioners caring for newborns at risk of hypoglycaemia in Aotearoa, New Zealand, both use and prefer accurate blood sampling devices, although this depended on the level of care.
  ○ Most respondents reported consistent timing of blood sampling, but there remained some variation.
  ○ These findings support the likelihood of successful implementation of a national guideline recommending accurate devices for blood sampling and standard timing of sampling.

## 1 Background

Neonatal hypoglycaemia is the most common metabolic disturbance in newborns and can lead to irreversible brain injury [1]. The incidence varies by population and testing protocol, affecting 40% of births and 27% of babies considered at risk [2, 3]. Managing this condition is crucial, yet uncertainties remain about how best to do this, including optimal testing methods.

One challenge is selecting appropriate testing devices, since inaccuracies can lead to over‐ and under‐diagnosis and consequent unnecessary intervention or missed diagnosis [4]. Many point‐of‐care devices provide rapid results and are easily accessed, but may be less accurate than standard laboratory methods, particularly at the lower blood glucose concentrations common in neonates, although this depends upon the specific device [4, 5, 6]. In contrast, laboratory analysers are usually more accurate but involve longer wait times, and variations in access to these testing devices may also contribute to inequity in healthcare. A recent systematic review reported that the three general analysis methods were most accurate in the measurement of neonatal blood glucose concentrations < 2.6 mmol/L: hexokinase + electrochemistry, glucose oxidase + electrochemistry, and glucose dehydrogenase + electrochemistry [7]. However, it is not always easy to determine which method is used in each specific device.

Clinical practise guidelines are essential for enhancing healthcare and improving patient outcomes. Previous research shows that following hypoglycaemia guidelines improves the detection of neonatal hypoglycaemia, even in asymptomatic, at‐risk newborns [8]. However, guidelines vary widely across hospitals and countries, highlighting the need for a national guideline based on the best available evidence on the management of neonatal hypoglycaemia [9, 10]. Developing guidelines without considering feasibility of implementation may hinder adherence [11]. Specifically, we reasoned that if the guidelines being developed recommended devices and processes already in use or preferred by practitioners, they are more likely to be implemented nationally than if new or less preferred practises were recommended.

Thus, we aimed to assess current testing practises for neonatal hypoglycaemia nationwide to inform recommendations for testing, including devices and timing, in the Te Tohu Waihonga Aotearoa New Zealand Clinical Practise Guideline for Neonatal Hypoglycaemia.

## 2 Materials and Methods

This study was reported according to the Checklist for Reporting of Survey Studies (CROSS) [12]. We sent invitation emails containing a link to an online survey created on the electronic platform Qualtrics to relevant professional groups across New Zealand's 19 health regions, including midwifery leaders, directors of midwifery, neonatal and maternity care units managers/leaders, and Maternity Quality and Safety Programme (MQSP) personnel or equivalent. Using Convenience sampling, recipients were asked to complete the survey themselves or identify the appropriate practitioner in their unit to respond. Additionally, an invitation was sent to lead maternity carers (LMC midwives; responsible for care throughout pregnancy, birth and the postnatal period and work across community and hospital settings) who provide home birth services through the New Zealand College of Midwives database. We did not calculate a formal sample size, aiming instead to gather insights from as many hospitals and care settings as possible to capture diverse perspectives. Respondents' multiple participation was prevented by IP address checking and follow‐up emails addressed non‐response error.

The cross‐sectional survey comprised 16 closed‐ended multiple‐choice questions about blood sampling devices used and preferred, the timing of sample collection, and a final open‐ended free‐text question for comments about testing for neonatal hypoglycaemia (see Supporting Information). Display logic functionality tailored the survey to each respondent, displaying only pertinent questions based on previous answers. Analyses were conducted using IBM SPSS version 28 [13]. Not all questions were compulsory, and some allowed multiple answers, leading to varying total response numbers. Numbers and proportions for categorical variables were calculated and compared between levels of care and between professions using chi‐square or Fisher's exact test with Bonferroni correction as needed to reduce Type I error risk in multiple pairwise comparisons. The level of care was determined by the name of the care unit or, for core midwives, the level of care facility at which they reported working most hours.

Participant consent was obtained on the first question of the survey. The Auckland Health Research Ethics Committee approved this study, reference number 25545. All survey data were confidential, accessible only to the research team, with no modifications to the original variables collected.

The analysis focused on available responses without imputing values or using methods like item weighting or propensity scores to adjust for non‐representativeness, and no specific confounders were assessed.

## 3 Results

### 3.1 Characteristics of Respondents

From 28th August to 12th December 2023, 71 participants responded to the survey. Respondents were from multiple professions and worked in all except one of New Zealand's health regions. The majority were core midwives who work with women in hospitals or birthing centres for their pregnancy, labour and postnatal needs, with the remaining being LMCs, neonatal/paediatric consultants, Directors/managers of care units and nurses (Table 1). ‘Other’ professions included educator, senior diabetes midwife, manager and associate director of midwifery. Of 68 respondents who stated their ethnicity with 54/68 (79.4%) were European, 4/68 (5.9%) Māori, 1/68 (1.5%) Chinese, and 13/68 (19.1%) ‘other’.

**TABLE 1:** Professions of the 71 respondents.

Profession	*n*	%^a^
Director/manager of the care unit	13	18.3%
Core midwife	30	42.3%
LMC/community midwife	19	26.8%
Registered nurse	2	2.8%
Neonatal/paediatric consultant	14	19.7%
Other (specify)	6	8.5%

^a^ Percentages are of the 84 responses: some respondents selected multiple professions.

Of the LMCs, 15/18 (83%) provided home birth services. Of the core midwives, the majority (17/29, 58.7%) worked most hours at secondary maternity facilities, with 6/29 (20.7%) working at primary birthing units and 6/29 (20.7%) working at tertiary maternity facilities.

### 3.2 Blood Glucose Concentration Sampling Devices

All but one respondent (69/70) stated that a capillary heel‐prick blood sample was used to screen for neonatal hypoglycaemia. The most common device used for analysing capillary blood samples was a blood gas analyser (20/65, 31%), followed by i‐STAT (12/65, 18%). A blood gas analyser was most commonly used for subsequent tests if the initial blood glucose measurement was low (19/57, 33%), followed by i‐STAT (13/57, 23%). For arterial or venous samples, the most common device used was a blood gas analyser (14/40, 35%) or an unknown method in a laboratory (13/40, 33%) (Table 2). Respondents who offer home birth services most commonly used i‐STAT (3/10, 30%) or ACCU‐CHEK (4/10, 40%) for all samples.

**TABLE 2:** Devices used by respondents for analysing neonatal blood samples.

Device	(A) Which methods are used for analysing capillary samples? [65 responses from 56 respondents]	(B) What method is used for analysing subsequent capillary samples if the initial blood glucose concentration is low? [57 responses from 46 respondents]	(C) Which methods are used for analysing arterial or venous samples? [40 responses from 29 respondents]^a^
Dextrostix	1	1	—
HemoCue	4	3	1
ACCU‐CHEK	10	8	1
FreeStyle NeoH	3	1	1
i‐STAT	12	13	6
Blood gas analyser (e.g., ABL 90, ABL 800)	20	19	14
Sending to the lab for analysis, instrument unknown	4	5	13
Super Glucocard 2	1	1	—
Enterprise point of care test	1	1	—
Don't know	4	3	3
Other	5	2	1

*Note:* Respondents could select multiple devices for each question.

^a^ Question not asked of those who offer home births.

Blood gas analysers were the most commonly used devices in tertiary units (8/12, 67%) and secondary units (10/35, 29%), and ACCU‐CHEK was the most commonly used device in primary units (6/11, 55%), with no use of blood gas devices reported from primary units. The range of devices used was not statistically different between tertiary and secondary units (*p* = 0.233) or secondary and primary units (*p* = 0.028) but did differ between tertiary and primary units (*p* = 0.002) (significance threshold 0.017 after Bonferroni correction for multiple comparisons) (Table 3).

**TABLE 3:** Devices used by respondents for analysing capillary samples against different levels of care.

Device	Tertiary unit [12 responses from 11 respondents]	Secondary unit [35 responses from 25 respondents]	Primary unit [11 responses from 9 respondents]
Dextrostix	1	—	—
HemoCue	—	4	—
ACCU‐CHEK	2	4	6
FreeStyle NeoH	—	2	1
i‐STAT	1	6	1
Blood gas analyser (e.g., ABL 90, ABL 800)	8	10	—
Sending to the lab for analysis, instrument unknown	—	3	—
Don't know	—	2	2
Other	—	4	1

*Note:* Some respondents selected multiple devices, 58 responses from 45 respondents. LMCs did not provide information about the unit level of care.

When asked about the device they would prefer to use for neonatal blood glucose testing, respondents chose a wide variety, with the most common choices being i‐STAT (22/80, 28%) and blood gas analyser (19/80, 24%) (Table 4). The preferred devices for core midwives were the i‐STAT (7/29, 24%), blood gas analyser (5/29, 17%) and ACCU‐CHEK (4/29, 14%). For LMCs, the most commonly identified device was i‐STAT (4/14, 29%), and for doctors, they were blood gas analyser (8/16, 50%) and i‐STAT (5/16, 31%). Managers of care units preferred i‐STAT (6/20, 30%), blood gas analyser (5/20, 25%) and ACCU‐CHEK (5/20, 25%) (Table 4). These differences between professions were not statistically significant (*p* = 0.141). The reasons given for the choice of testing devices were accuracy, ease of device use, access, and speed of receiving results.

**TABLE 4:** Devices preferred, resources permitted, by respondents for analysing neonatal blood samples.

Preferred device	Core midwives [29 responses from 21 respondents]	LMC [14 responses from 10 respondents]	Neonatal/paediatric consultant [16 responses from 10 respondents]	Director/manager of the care unit [20 responses from 12 respondents]	Nurse [1 respondent]	Total [80 responses from 49 respondents]^a^
Dextrostix	2	—	—	—	—	2
HemoCue	1	—	2	1	—	4
ACCU‐CHEK	4	1	—	5	1	11
FreeStyle NeoH	1	1	1	—	—	3
i‐STAT	7	4	5	6	—	22
Blood gas analyser (e.g., ABL 90, ABL 800)	5	1	8	5	—	19
Sending to the lab for analysis, instrument unknown	—	1	—	—	—	1
Elite XL	1	—	—	—	—	1
Enterprise point of care (EPOC) test	2	—	—	2	—	4
Other or not known	6	6	—	1	—	13

^a^ Total number of respondents does not reflect the other column totals because responses from respondents who selected multiple professions are included under each profession.

### 3.3 Reported Procedures for Sample Collection

Most respondents (45/59, 76%) reported that the first blood sample should be taken between 1 and < 2 h after birth, but others (5/59, 8.5%) reported that this should be within the first hour, between 2 and 4 h (4/59, 6.8%) or should be timed relative to feeds (5/59, 8.5%). Most respondents (41/58, 71%) reported that three tests should be taken in babies with normal blood glucose concentrations, but others reported four tests or that the tests should be timed pre‐feeding or fasting (17/58, 29%). Most (46/56, 82%) also reported that the repeat blood glucose test should be done 30 min after treatment was given, whereas others stated that this should be done between 30 and < 60 min (4/56, 7.1%) or ≥ 120 min after (1/56, 1.8%) the initial blood glucose measurement was taken. However, others were less clear (5/56, 8.9%), with one reporting that their guideline did not specify and another being unsure.

Most respondents (35/59, 59%) reported the blood test results were available within 1 min, and 32% (19/59) reported this was within 5 min, but 5% (3/59) reported that the test results were available within 10 min.

## 4 Discussion

This study provided a snapshot of the processes related to testing for neonatal hypoglycaemia across New Zealand. Most respondents were midwives, who provide most primary newborn care in New Zealand [14]. Blood gas analysers and i‐STAT devices were the most common blood sampling devices used by participants, and this did not differ for different professions, but did differ between levels of care, with no blood gas analyser use reported from primary units. Respondents most often reported that they would prefer to use blood gas analysers and i‐STAT devices because of perceived accuracy, accessibility, and speed of results. There was a range of reported timing of blood samples and how many should be taken. Over half of the respondents stated that blood sample results were available within 1 min.

The blood gas analyser was the most used device for capillary blood samples, subsequent tests if the initial screening test result was low, and for measuring arterial or venous samples. This is reassuring as most blood gas analysers use reliable glucose oxidase or hexokinase methods for glucose measurement [15]. In contrast, a New Zealand survey in 2014 raised concerns about the point‐of‐care glucose testing devices used to diagnose neonatal hypoglycaemia, noting that these devices, whereas convenient, were often used in secondary facilities and could lead to inaccuracies with both overestimation and underestimation of glucose concentrations [15]. Our survey found that blood gas analysers are now the devices most commonly used by respondents working primarily in secondary units, indicating a shift in practise since the 2014 survey and increased availability of blood gas analysers for these secondary units. This trend was the same for tertiary units, with 67% (8/12) using blood gas analysers. However, primary units mainly used ACCU‐CHEK (6/11, 55%), with none reporting use of blood gas analysers, suggesting potential gaps in access and availability for these units. The authors of the 2014 paper recommended that point‐of‐care devices should only be used as a screening tool, with confirmation done using a reliable device [15].

With the continuing development of new devices and methods to detect neonatal blood glucose concentrations, it can be hard to assess what device is reliable. A recent systematic review and meta‐analysis found three groups of devices were most accurate for the measurement of neonatal blood glucose concentrations < 2.6 mmol/L: those using hexokinase + electrochemistry, glucose oxidase + electrochemistry, and glucose dehydrogenase + electrochemistry [7]. This is promising as both the most commonly used devices: blood gas analyser and i‐STAT, use these methods [15]. However, it was not always possible to determine which device and method were being reported in this survey. For example, many respondents reported using ACCU‐CHEK, but many different models and methods are included in this brand name, so it is difficult to determine whether more or less accurate models were used. Nevertheless, even if all responses naming “ACCU‐CHEK” were assumed to refer to an accurate device, 83.3% (50/60) of respondents currently used these devices for the first blood sample analysis, which meant that 16.7% (10/60) still either did not know or used less accurate devices, and this was true for 14.5% (8/55) for subsequent tests when the initial result is low [16]. Promisingly, most respondents (61/67, 91%) indicated accurate blood sampling devices when asked about the device they would prefer to use, and this was true across all professional groups. Regularly updating a widely available summary of the accuracy of available devices for neonatal blood glucose measurements would help support best practise across all settings that care for newborn babies.

There was reasonable agreement about the timing of testing for hypoglycaemia, with > 70% of respondents selecting the same timings for the first blood sample, the second sample for a baby with hypoglycaemia, and the total number of blood samples needed. However, others selected different timings, highlighting the limited evidence available to guide decision‐making, which results in decisions based on the judgement of individual clinical units in different parts of New Zealand. Similar variation is seen internationally, with guidelines from the American Academy of Paediatrics, Paediatric Endocrine Society, Canadian Paediatric Society, British Association of Perinatal Medicine, and the European Foundation for the Care of the Newborn Infants making recommendations for the timing of the first blood sample in at‐risk infants ranging from before the first feed to 2 h of age to before the second feed (first 2–4 h) [17, 18, 19, 20].

The variations in practise reported across different health regions and professions may, in part, be due to access to different testing devices. This is likely to be affected by established practise and also cost, particularly as LMCs do not have access to dedicated funding for testing devices. The i‐STAT and ACCU‐CHEK were the devices most commonly used by those providing home birth services. This is consistent with the lack of availability of blood gas analysers in home birth settings, and potentially also in primary units. Many respondents expressed a preference for using the most accurate and reliable device, suggesting that health provider attitudes are unlikely to hinder the implementation of recommended devices. However, funding and access to these devices may be limiting, especially for those working in the community‐based setting, and this may contribute to inequity in healthcare.

A strength of this survey was that it was created on a widely recognised online survey generator, Qualtrics, which allowed display logic functionality to facilitate the presentation of straightforward and profession‐specific questions. Respondents were also from a range of professions across almost all regions, so our findings likely apply to healthcare settings across New Zealand.

However, this survey relied on respondents to recall information and accurately describe processes used for testing neonatal hypoglycaemia, introducing a potential for recall bias. The sample was also not chosen at random, with unit leaders being contacted and relied upon to select the best respondents. This method may have been a limitation in capturing all different types of practise in New Zealand, but did result in responses from across the range of birthing facilities used in New Zealand (46% tertiary, 42% secondary, 8% primary, 4% home in 2022 [21]). Another limitation was the inability to calculate the response rate, as we had no way of knowing how many invitations were sent by the relevant professional groups. Additionally, since the questions were optional, not all 74 respondents answered every question. We also lacked responses from other groups of providers potentially involved in decisions about neonatal hypoglycaemia testing, such as laboratory managers, obstetricians, and point‐of‐care managers.

## 5 Conclusion

Most of the healthcare professionals caring for newborns at risk of hypoglycaemia use accurate blood sampling devices and prefer these devices, independent of profession. This suggests the implementation of a national recommendation to use only accurate devices is not likely to be resisted by clinician preference. However, access and cost constraints may pose greater barriers to implementation. Additionally, there is a need for consistent guidance surrounding the timing of testing for neonatal hypoglycaemia.

## Ethics Statement

This study was approved by the Auckland Health Research Ethics. Reference number: 25545.

## Consent

Participant consent was obtained on the first question of the online survey.

## Conflicts of Interest

The authors declare no conflicts of interest.

## Supporting information


**Data S1.** jpc70097‐sup‐0001‐Supinfo1.
