# Supplementary material for: A Survey of the Current Practice for Testing for Neonatal Hypoglycaemia in Aotearoa, New Zealand
Source: J Paediatr Child Health. 2025 May 31;61(8):1235–40. doi: 10.1111/jpc.70097 (PMC12397843; doi:10.1111/jpc.70097)
Supplement: Supplementary file 1 — Data S1. jpc70097‐sup‐0001‐Supinfo1. [file JPC-61-1235-s001.docx]

**Supporting information: Qualtrics survey**

**Consent: Thank you for taking the time to participate in this survey. By selecting "I agree", your informed consent will be given and the survey will start:**

o I agree

o I don't agree

**Q1. What is your profession (Tick as many as apply)**

▢ Director/ manager of the care unit

▢ Core Midwife

▢ LMC/community midwife

▢ Registered Nurse

▢ Neonatal Nurse Practitioner

▢ Neonatal/paediatric consultant

▢ Neonatal Medical Officer of Special Scale (MOSS)

▢ Obstetric consultant

▢ Obstetric Medical Officer of Special Scale (MOSS)

▢ Laboratory manager

▢ Point of care manager

▢ Other (specify) **__________________________________________________**

*Display This Question:*

*If 1. What is your profession (Tick as many as apply) = LMC/community midwife*

**Q1a. Do you offer home birth?**

o Yes

o No

*Display This Question:*

*If 1. What is your profession (Tick as many as apply) = Core Midwife*

**Q1b. Where do you work the most hours? (Tick one)**

o Primary birthing unit

o Secondary maternity facility

o Tertiary maternity facility

**Q2. Which ethnic group do you belong to? Select the option or options that apply to you.**

▢ Māori

▢ Samoan

▢ Cook Island Māori

▢ Tongan

▢ Niuean

▢ Chinese

▢ Indian

▢ New Zealand European

▢ Other (specify: such as Dutch, Japanese, Tokelauan) **___________________________**

**Q3. In which DHB region do you work? (If more than one, please name the unit where you work most often)**

o Te Whatu Ora Te Tai Tokerau (formerly Northland District Health Board)

o Te Whatu Ora Waitematā (formerly Waitemata District Health Board)

o Te Whatu Ora Te Toka Tumai Auckland (formerly Auckland District Health Board)

o Te Whatu Ora Counties Manukau (formerly Counties-Manukau District Health Board)

o Te Whatu Ora Waikato (formerly Waikato District Health Board)

o Te Whatu Ora Hauora a Toi Bay of Plenty (formerly Bay of Plenty District Health Board)

o Te Whatu Ora Lakes (formerly Lakes District Health Board)

o Te Whatu Ora Tairāwhiti (formerly Tairawhiti District Health Board)

o Te Whatu Ora Taranaki (formerly Taranaki District Health Board)

o Te Whatu Ora Whanganui (formerly Whanganui District Health Board)

o Te Whatu Ora Te Pae Hauora o Ruahine o Tararua MidCentral (formerly MidCentral District Health Board)

o Te Whatu Ora Te Matau a Māui Hawke's Bay (formerly Hawke's Bay District Health Board)

o Te Whatu Ora Wairarapa (formerly Wairarapa District Health Board)

o Te Whatu Ora Capital, Coast and Hutt Valley (formerly Capital & Coast District Health Board, and Hutt Valley District Health Board)

o Te Whatu Ora Nelson Marlborough (formerly Nelson-Marlborough District Health Board)

o Te Whatu Ora Te Tai o Poutini West Coast (formerly West Coast District Health Board)

o Te Whatu Ora Waitaha Canterbury (formerly Canterbury District Health Board)

o Te Whatu Ora South Canterbury (formerly South Canterbury District Health Board)

o Te Whatu Ora Southern (formerly Southern District Health Board)

*Display This Question:*

*If 1. What is your profession (Tick as many as apply) != LMC/community midwife*

**Q4. What is the name of your care unit (e.g. Labour and Birthing suite, Te Toka Tumai Auckland)?**

________________________________________________________________

**Q5. Which kind of blood sample is most commonly taken for the screening for neonatal hypoglycaemia in your hospital/setting? (Tick one)**

o Capillary heel-prick blood sample

o Venous

o Arterial

o Other (please specify) __________________________________________________

*Display This Question:*

*If Do you offer home birth? != Yes*

**Q6a.** Which methods are used for analysing samples?

|  | Which methods are used for analysing capillary samples? (Tick all used in your setting) | What method is used for analysing subsequent capillary samples if the initial blood glucose concentration is low? (Tick all used in your setting) | Which methods are used for analysing arterial or venous samples? (Tick all used in your setting) |
| --- | --- | --- | --- |
| - Dextrostix |  |  |  |
| - Reflolux |  |  |  |
| - HemoCue |  |  |  |
| - Super Glucocard 2 |  |  |  |
| - Elite XL |  |  |  |
| - Precision G |  |  |  |
| - Advantage |  |  |  |
| - Glucotrend |  |  |  |
| - ACCU-CHEK^®^ |  |  |  |
| - FreeStyle NeoH |  |  |  |
| - i-STAT^TM^ |  |  |  |
| - Enterprise Point of Care (EPOC) test |  |  |  |
| - Blood gas analyser (e.g. ABL 90, ABL 800) |  |  |  |
| - Yellow Springs Instruments analyser (YSI) |  |  |  |
| - Sending to the lab for analysis, instrument unknown |  |  |  |
| - Don't know |  |  |  |
| - Other (please specify) |  |  |  |

*Display This Question:*

*If Do you offer home birth? = Yes*

**Q6b.** Which methods are used for analysing samples?

|  | Which methods are used for analysing capillary samples? (Tick all used in your setting) | What method is used for analysing subsequent capillary samples if the initial blood glucose concentration is low? (Tick all used in your setting) |
| --- | --- | --- |
| Dextrostix |  |  |
| Reflolux |  |  |
| HemoCue |  |  |
| Super Glucocard 2 |  |  |
| Elite XL |  |  |
| Precision G |  |  |
| Advantage |  |  |
| Glucotrend |  |  |
| ACCU-CHEK^®^ |  |  |
| FreeStyle NeoH |  |  |
| i-STAT |  |  |
| Enterprise Point of Care (EPOC) test |  |  |
| Blood gas analyser (e.g. ABL 90, ABL 800) |  |  |
| Yellow Springs Instruments analyser (YSI) |  |  |
| Sending to the lab for analysis, instrument unknown |  |  |
| Don't know |  |  |
| Other (please specify) |  |  |

**Q7. How are the results recorded (Tick as many as apply)?**

▢ Electronic clinical records

▢ Paper clinical records

▢ Bedside/nursing records

▢ Don’t know

▢ Other (please specify) __________________________________________________

**Q8. In babies who are having blood tests to detect low glucose concentrations (hypoglycaemia), when does the guideline in use at your hospital/setting recommend the first sample is taken? Or when is the first sample usually taken if there is not a guideline to follow? (Tick one)**

o < first 30 minutes after birth

o < first hour after birth

o Between 1 and < 2 hours after birth

o Between 2 and < 4 hours after birth

o Before the first feed

o Before the second feed

o Other (please specify) __________________________________________________

**Q9. How long does it usually take after sample collection before the results are available? (Tick one)**

o < 1 minute

o < 5 minutes

o < 10 minutes

o Between 10 and < 20 minutes

o Between 20 and < 30 minutes

o Between 30 and < 60 minutes

o ≥ 60 minutes

**Q10. How many blood glucose measurements does your local guideline recommend (or are supposed to be taken) in at-risk babies if the results are all normal > 2. 6 mmol/L? (Tick one)**

o One

o Two

o Three

o Other (please specify) __________________________________________________

**Q11. For babies with hypoglycaemia on the initial blood glucose sample, when does the guideline in use at your hospital/setting recommend repeating the blood glucose concentration (or when is it supposed to be done)? (Tick one)**

o 30 minutes after treatment was given

o < 30 minutes after the initial blood glucose was taken

o Between 30 to < 60 minutes after the initial blood glucose was taken

o Between 60 to < 90 minutes after the initial blood glucose was taken

o Between 90 to < 120 minutes after the initial blood glucose was taken

o ≥120 minutes after the initial blood glucose was taken

o Other (please specify) __________________________________________________

**Q12. If resources permitted, which methods would you prefer to use for testing neonatal hypoglycaemia? (Tick as many as apply)**

▢ Dextrostix

▢ Reflolux

▢ HemoCue

▢ Super Glucocard 2

▢ Elite XL

▢ Precision G

▢ Advantage

▢ Glucotrend

▢ ACCU-CHEK^®^

▢ FreeStyle NeoH

▢ i-STAT

▢ Enterprise Point of Care (EPOC) test

▢ Blood gas analyser (e.g. ABL 90, ABL 800)

▢ Yellow Springs Instruments analyser (YSI)

▢ Sending to the lab for analysis, instrument unknown

▢ Other (please specify) __________________________________________________

**Q13. What are the reasons for your preferred choice of methods?**

________________________________________________________________

**Q14. Do you have any other comments about testing for neonatal hypoglycaemia?**

________________________________________________________________
